# Distributed formation for interconnected networks with minimum energy restriction and interaction silence

**DOI:** 10.1038/s41598-023-31244-0

**Published:** 2023-03-12

**Authors:** Yukun Qiao, Junlong Li, Qi Zhang, Jianxiang Xi, Yuanshi Zheng

**Affiliations:** 1 School of Electronic Information, Xijing University, Xi’an, 710123 People’s Republic of China; 2 School of Missile Engineering, PLA Rocket Force University of Engineering, Xi’an, 710025 People’s Republic of China; 3 Lüliang University, Lüliang, 033001 People’s Republic of China; 4 Shaanxi Key Laboratory of Space Solar Power Station System, School of Mechano-Electronic Engineering, Xidian University, Xi’an, 710071 People’s Republic of China

**Keywords:** Computer science, Electrical and electronic engineering

## Abstract

The minimum-energy formation strategy for interconnected networks with distributed formation protocols is persented, where the impacts of the total energy restriction and the interaction silence are analyzed, respectively. The critical feature of this article is that the distributed formation and the minimum-energy restriction are realized simultaneously, and the total energy restriction is minimum in the sense of the linear matrix inequality. However, the guaranteed-cost formation strategy and the limited-budget formation strategy cannot guarantee that the energy restriction is minimum. Firstly, sufficient conditions for minimum-energy-restriction formation without the interaction silence are proposed, which can be solved by a specific optimization approach in terms of the linear matrix inequality, and the formation whole motion trajectory is determined, which is closely related to the average of the initial states of all agents and formation control vectors. Then, minimum-energy-restriction formation criteria for interconnected systems with the interaction silence are proposed by introducing two inhibition parameters and the interaction silence rate. Finally, two simulation examples are performed to illustrate the effectiveness of theoretical analyses.

## Introduction

The cooperative behaviors of interconnected systems attracted much interests in various research fields, such as the flocking behavior^1,2^, the containment behavior^3^, the consensus behavior^4–6^, the coordinated fusion behavior^7–9^, and the formation behavior^10–12^, *et al*. Especially, the investigation of the formation behavior of interconnected systems inspires by the distributed mechanism research of the flocking and consensus behaviors, where the flocking and consensus behaviors mainly focus on the theoretical analysis, but the formation behavior has extensive applications in practical interconnected systems, such as the coordinated attack of multiple drones and the cooperative operation of multiple intelligent robots.

It is necessary for distributed formation control that all agents maintain a specific structure to finish some tasks via a partial information interaction, as shown in^13–17^. Furthermore, the energy restriction is a necessary component for practical applications of interconnected systems and can be categorized into two types: a local energy restriction and a total energy restriction. Semsar-Kazerooni and Khorasani^18^ proposed a local restriction function on the basis of the partial information interaction and realized an optimal cooperation. Vamvoudakis *et al*.^19^ presented a multi-agent differential graphical game approach and gave an online adaptive learning solution with a local restriction function. A total restriction function associated with the linear quadratic regulator was proposed in^20^, where the optimal cooperation can be achieved when the information topology is complete. In^21–23^, the guaranteed-cost strategy was proposed, where the different upper bounds of the total energy restriction were obtained, but the error between these bounds and the minimum bound cannot be determined. The limited-budget strategy was presented in^24^, where an upper bound of the energy restriction was specified in advance. However, these two upper bounds of the total energy restriction may not be the minimal value, and both the guaranteed-cost and limited-budget formation strategies cannot guarantee that the total energy restriction is minimum.

Moreover, in the above literatures on distributed formation control with the energy restriction, it was required that the information topology is connected or has a spanning tree and must maintain the continuous transmission of cooperative information. However, there may be instances where the information transmission fails due to the channel jamming or the anti-jamming. Information interactions of interconnected systems may disappear and recover when there is the electronic interference in the communication environment. Furthermore, the active silence strategies may be adopted by the interconnected system to save the energy or to avoid the detection of some electromagnetic spectrum equipment. In this case, there exists no information interaction among agents in some time segments. Qin *et al*.^25^ proposed an observer-based formation strategy that takes into account the interaction silence. For heterogeneous linear interconnected systems, sufficient conditions for output formation-containment were presented in^26^, where an approach was proposed to optimize the interaction silence rate. Yang *et al*.^27^ explored the adaptive control strategy for interconnected systems with the interaction silence and theoretical results were applied to cooperative teleoperators. Wang *et al*.^28^ established a new interaction silence framework for heterogeneous coupling networks using the linear matrix inequality technology. With the interaction silence, it is meaningful and challenging to minimize the total energy restriction of interconnected networks by regulating the control gain matrix of the distributed formation protocol. To the best of our knowledge, distributed formation control problems for interconnected systems with the minimum energy restriction and the interaction silence are not comprehensively investigated and are still open.

This article focuses on minimum-energy-restriction formation achievement problems with the interaction silence in interconnected systems. A distributed formation protocol is shown to realize minimum-energy-restriction formation control, where a total energy restriction term is imposed. Furthermore, sufficient conditions for minimum-energy-restriction formation without the interaction silence are proposed by establishing the relationship between the energy restriction and the gain matrix. These conditions are scalable since the dimension of the matrix variable is not related to the number of agents and can be verified by the generalized eigenvalue method. Especially, the formation whole motion trajectory is obtained, where the effects of the initial states and formation control vectors are depicted, respectively. Moreover, interconnected systems with the interaction silence are dealt with, where two inhibition parameters and the interaction silence rate are introduced to restrict the divergent quantity during the interaction silence subsegment, and the associated criteria are proposed to realize formation control with the minimum energy restriction. It should be pointed out that the interaction silence is essentially the same as the intermittent control from the point of the information interaction in interconnected networks. However, from the point of the control method, some event-triggered control strategies can also lead to the intermittent control.

Compared with the existing literature of optimization formation, the contributions and innovations of this article own the following three novel aspects. The first aspect is that this article realizes the distributed formation control and the minimum-energy restriction simultaneously. However, the guaranteed-cost strategy and the limited-budget strategy in^22–24^ cannot minimize the total energy restriction of all agents as a whole. The second aspect is that the effects of the interaction silence on the formation control with the minimum-energy restriction are handled by introducing two inhibition parameters and the interaction silence rate in this article, and a specific condition about the interaction silence rate is constructed to constrain the divergent quantity during the interaction silence subsegment. However, the influence of the interaction silence on the minimum energy restriction was not considered in^22–24^. The third aspect is that the explicit expression of the whole motion trajectory for the minimum-energy-restriction formation is determined, where the effects of the initial states of all agents and formation control vectors are depicted and it is shown that the interaction silence does not impact the formation whole motion trajectory, which were not settled in^22–24^.

The rest sections are to be outlined as follows. Without the interaction silence, the problem statement for formation control of interconnected systems with the minimum energy restriction is presented in “Problem statements for minimum-energy-restriction formation” section. Based on the strategy of the linear matrix inequality, “Minimum-energy-restriction formation without interaction silence” section proposes sufficient conditions for the minimum-energy-restriction formation without the interaction silence. In “Minimum-energy-restriction formation with interaction silence” section, the effects of the interaction silence are investigated and the associated criteria for the minimum-energy-restriction formation are shown. “Simulation examples” section gives two examples to check the impacts of the energy restriction and the interaction silence, respectively. The whole work is summarized in “Conclusions” section.

The key notations used in this work are shown in the following Table 1.

**Table 1:** Notation index.

Notations	Implication
*M*	Number of agents
*d*	Dimension of cooperative state
$\mathbb {R}$	Real number set
${\bar{\mathbb {Z}}^ - }$	Nonnegative integer number set
$\xi (t)$	Formation function
*G*	Information topology
*L*	Laplacian matrix
${\textbf{1}}$	Column vector with all components 1
${\text {0}}$	Zero number
${\textbf{0}}$	Zero matrix
$\otimes$	Kronecker product
$\varpi$	Interaction silence rate
$*$	Symmetric term in a symmetric matrix

## Problem statements for minimum-energy-restriction formation

Consider an interconnected system with *M* homogenous agents as follows:

1 $$\begin{aligned} {\dot{x}_i}(t) = A{x_i}(t) + B{u_i}(t)\,\,\left( {i = 1,2, \ldots ,M} \right) , \end{aligned}$$

where $A \in {\mathbb {R}^{d \times d}}$, $B \in {\mathbb {R}^{d \times m}}$, ${x_i}(t)$ is the cooperative state, and ${u_i}(t)$ is the formation protocol. Furthermore, to realize the distributed formation control, the formation control vector of each agent is time-varying; that is, there exist *M* vectors ${\xi _i}(t) \in {\mathbb {R}^d}$ $\left( {i = 1,2, \ldots ,M} \right)$ to realize the desired formation, which is time-varying and piecewise continuous differentiable. Note that ${\xi _i}(t) \in {\mathbb {R}^d}$ $\left( {i = 1,2, \ldots ,M} \right)$ can define arbitrary formation structures, but the feasibility of these structures is constrained by the dynamics of each agent, which intrinsically reflects the restriction of the physical structure of practical systems. Moreover, if all formation control vectors are time-invariant, then the corresponding formation structure is time-invariant; that is, interconnected system (1) is needed to achieve a static formation. Otherwise, a time-varying formation must be obtained.

The information topology of this interconnected system is described by a graph *G*, where the vertex in the graph stands for an individual agent, its edge represents an interconnected channel and its edge weight denotes the channel strength between two neighboring agents. The information topology of interconnected systems is undirected and connected, which means that two neighboring agents can acquire the cooperative information with each other simultaneously and there does not exist an agent unconnected with other agents. Especially, there is not a self-loop in this information topology *G*, which means that each agent can acquire its cooperative information directly without any communication actions. If agent *i* $\left( {i \in \{ 1,2, \ldots ,M \}} \right)$ can acquire the cooperative information of agent *k* $\left( k \in \{ 1,2, \ldots ,M \} \right)$, then agent *k* is called a neighbor of agent *i*. The set containing all neighbors of agent *i* is called its neighbor set, which is denoted by ${\mathcal {N}_i}$. The mathematic characteristics of this information topology can be depicted by Laplacian matrix *L*, whose row sum is zero as shown in^29^; that is, $L{\textbf{1}} = {\textbf{0}}$. It is assumed that the information topology satisfied:

### Assumption 1

The information topology in this article is undirected and connected.

The formation protocol of each agent is usually composed of the cooperative states and the formation control vectors of neighboring agents of each agent. We construct a formation protocol with the energy restriction as follows:

P1 $$\begin{aligned} \left\{ \begin{array}{ll} {u_i}(t) = K\sum \limits _{k \in {\mathcal {N}_i}} {{w_{ik}}\left( {{x_k}(t) - {\xi _k}(t) - {x_i}(t) + {\xi _i}(t)} \right) } , \\ E = \sum \limits _{i = 1}^M {\int _0^{ + \infty } {u_i^T(t)W{u_i}(t){\text {d}}t} } , \\ \end{array}\right. \end{aligned}$$

where $i = 1,2, \ldots ,M,$ ${W^T} = W > 0$ is a weighted matrix, ${w_{ik}}$ is the channel strength between agents *i* and *k*, $K \in {\mathbb {R}^{m \times d}}$ is a gain matrix, and the scalar *E* is the overall energy restriction. This formation protocol is distributed since each agent regulates its cooperative state by synthesizing the cooperative information of its neighboring agents and all agents have the same cooperative control gain. The following is the definition of the minimum-energy-restriction formation.

### Definition 1

For any bounded disagreement initial conditions ${x_i}(0) - {\xi _i}(0)$ $\left( {i = 1,2, \ldots ,M} \right)$, interconnected system (1) is said to be *minimum-energy-restriction formation achievable* by formation protocol (P1) if there exists a gain matrix *K* such that ${\lim _{t \rightarrow + \infty }}\left( {{x_k}(t) - {x_i}(t)} \right.$ $\left. { - {\xi _k}(t) + {\xi _i}(t)} \right) = {\textbf{0}}$ $\left( {\forall i,k \in \left\{ {1,2, \ldots ,M} \right\} } \right)$ and the energy restriction is minimum.

The objective of this article is to design the cooperative control gain such that interconnected system (1) achieves formation with the minimum energy restriction and to propose sufficient conditions for minimum-energy-restriction formation design and analysis, respectively. Furthermore, the effects of the interaction silence on the formation with the minimum energy restriction are investigated and the associated minimum-energy-restriction formation criteria are proposed.

### Remark 1

There are two main types of control strategies that are commonly used for controlling the formation of the interconnected system with the total energy restriction; that is, the guaranteed-cost strategy and the limited-budget strategy, which were addressed in^23^ and^24^, respectively. For the formation control with the guaranteed-cost strategy, it is needed to give the upper bound of the guaranteed cost and the conservatism of this bound cannot be determined since it is difficult to calculate the error between this bound and the minimum bound. Although an upper bound is previously given for the limited-budget formation control, it cannot be guaranteed that this value of the upper bound is minimum. It is interesting to minimize the total energy restriction and to decrease the energy assumption of all agents as a whole. To make the energy restriction be minimum when formation is achieved, we need to deal with two key challenges. The first challenge in the formation process is to construct the relationship of the energy restriction and the gain matrix, which means that the energy restriction can be regulated by designing the different gain matrices. The second challenge is to develop a specific optimization approach such that the total energy restriction is minimum. This article focuses on minimizing the total energy restriction using the linear matrix inequality strategy.

## Minimum-energy-restriction formation without interaction silence

In this section, the relationship between the energy restriction and the gain matrix is constructed by the matrix variable of the Lyapunov function. Then, sufficient conditions for minimum-energy-restriction formation design and analysis are proposed, respectively, which can be obtained by a specific optimization approach.

Let ${\zeta _i}(t) = {x_i}(t) - {\xi _i}(t)\,\,(i = 1,2, \ldots ,M),$ then one can deduce by (1) and (P1) that

2 $$\begin{aligned} \left\{ \begin{array}{ll} {{\dot{\zeta }}_i}(t) = A{\zeta _i}(t) + A{\xi _i}(t) - {{\dot{\xi }}_i}(t) + B{u_i}(t), \\ {u_i}(t) = K\sum \limits _{k \in {\mathcal {N}_i}} {{w_{ik}}\left( {{\zeta _k}(t) - {\zeta _i}(t)} \right) } . \\ \end{array} \right. \end{aligned}$$

One can set that

$$\begin{aligned} \zeta (t) = {\left[ {\zeta _1^T(t),\zeta _2^T(t), \ldots ,\zeta _M^T(t)} \right] ^T}, \\ \xi (t) = {\left[ {\xi _1^T(t),\xi _2^T(t), \ldots ,\xi _M^T(t)} \right] ^T}, \end{aligned}$$

then interconnected system (2) can be rewritten in a Kronecker product form as

3 $$\begin{aligned} \dot{\zeta }(t) = \left( {{I_M} \otimes A - L \otimes BK} \right) \zeta (t) + \left( {{I_M} \otimes A} \right) \xi (t) - \dot{\xi }(t). \end{aligned}$$

The information topology is undirected and connected. Thus, all eigenvalues of *L* are real and there exists an orthogonal matrix *U* such that

$$\begin{aligned} {U^T}LU = {\text {diag}}\left\{ {{\lambda _1},{\lambda _2}, \ldots ,{\lambda _M}} \right\} , \end{aligned}$$

where $0 = {\lambda _1} < {\lambda _2} \leqslant \cdots \leqslant {\lambda _M}$. Due to $L{\textbf{1}} = {\textbf{0}}$, one can obtain that the first column of *U* is ${{\textbf{1}} / {\sqrt{M} }}$; that is, $U{e_1} = {{\textbf{1}} /{\sqrt{M} }}$, where ${e_1}$ denotes an *M*-dimensional column vector with the first component 1 and 0 elsewhere. Since *U* is orthogonal, one has

$$\begin{aligned} \left( {{U^T} \otimes {I_d}} \right) \zeta (t) = {\left[ {{\tilde{\zeta }} _1^T(t),{\tilde{\zeta }} _2^T(t), \ldots ,{\tilde{\zeta }} _M^T(t)} \right] ^T}. \end{aligned}$$

Thus, interconnected system (3) is transformed as

4 $$\begin{aligned} {\dot{{\tilde{\zeta }}} _1}(t)&= A{{\tilde{\zeta }} _1}(t) + \left( {e_1^T{U^T} \otimes A} \right) \xi (t) - \left( {e_1^T{U^T} \otimes {I_d}} \right) \dot{\xi }(t), \end{aligned}$$

5 $$\begin{aligned} {\dot{{\tilde{\zeta }}} _i}(t)&= \left( {A - {\lambda _i}BK} \right) {{\tilde{\zeta }} _i}(t) + \left( {e_i^T{U^T} \otimes A} \right) \xi (t) - \left( {e_i^T{U^T} \otimes {I_d}} \right) \dot{\xi }(t), \end{aligned}$$

where $$i = 2,3, \ldots ,M$$.

According to the above state decomposition and the linear matrix inequality technology, a sufficient condition for the formation achievement with the minimum energy restriction is presented by introducing the formation feasible condition. In the linear matrix inequality sense, the minimum energy restriction can be guaranteed and can be solved through the generalized eigenvalue optimization approach.

### Theorem 1

Interconnected system (1) is minimum-energy-restriction formation achievable by formation protocol (P1) with $K = \lambda _2^{ - 1}{B^T}{P^{ - 1}}$ if $A{\xi _i}(t) = {\dot{\xi }_i}(t)$ $\left( {i = 1,2, \ldots ,M} \right)$ and there exists ${P^T} = P > 0$ such that the following minimization problem has an optimal solution $\eta$:

$$\begin{aligned} \hbox {min } & \quad \eta \\ \hbox {s. t.} & \quad {\Omega _1} = \left[ {\begin{array}{*{20}{c}} {P{A^T} + AP - 2B{B^T}}&{\lambda _2^{ - 1}{\lambda _M}BW} \\ * &{ - W} \end{array}} \right]< 0,\\ & \quad {\Omega _2} = {I_d} - \eta P < 0. \end{aligned}$$

### Proof

Let

6 $$\begin{aligned}&{{\tilde{\zeta }} _w}(t) \triangleq \left( {U \otimes {I_d}} \right) {\left[ {{\tilde{\zeta }} _1^T(t),{\textbf{0}}, \ldots ,{\textbf{0}}} \right] ^T}, \end{aligned}$$

7 $$\begin{aligned}&{{\tilde{\zeta }} _r}(t) \triangleq \left( {U \otimes {I_d}} \right) {\left[ {{\textbf{0}},{\tilde{\zeta }} _2^T(t), \ldots ,{\tilde{\zeta }} _M^T(t)} \right] ^T}, \end{aligned}$$

then one can show that

8 $$\begin{aligned} \zeta (t) = {{\tilde{\zeta }} _w}(t) + {{\tilde{\zeta }} _r}(t). \end{aligned}$$

By ${\left[ {{\tilde{\zeta }} _1^T(t),{\textbf{0}}, \ldots ,{\textbf{0}}} \right] ^T} = {e_1} \otimes {{\tilde{\zeta }} _1}(t)$ and $U{e_1} = {{\textbf{1}} / {\sqrt{M} }}$, it can be derived via (6) that

9 $$\begin{aligned} {{\tilde{\zeta }} _w}(t) = \frac{1}{{\sqrt{M} }}{\textbf{1}} \otimes {{\tilde{\zeta }} _1}(t). \end{aligned}$$

Due to

$$\begin{aligned} {\left[ {{\textbf{0}},{\tilde{\zeta }} _2^T(t), \ldots ,{\tilde{\zeta }} _M^T(t)} \right] ^T} = \sum \limits _{i = 2}^M {{e_i} \otimes {{{\tilde{\zeta }} }_i}(t)}, \end{aligned}$$

one has

10 $$\begin{aligned} {\zeta _r}(t) = \sum \limits _{i = 2}^M {U{e_i} \otimes {{{\tilde{\zeta }} }_i}(t)} . \end{aligned}$$

By (6) and (7), ${{\tilde{\zeta }} _w}(t)$ and ${{\tilde{\zeta }} _r}(t)$ are linearly independent since *U* is orthogonal. Due to

$$\begin{aligned} \zeta (t) = \left( {U \otimes {I_d}} \right) {\left[ {{\tilde{\zeta }} _1^T(t),{\tilde{\zeta }} _2^T(t), \ldots ,{\tilde{\zeta }} _M^T(t)} \right] ^T}{{ = }}{\zeta _w}(t) + {\zeta _r}(t), \end{aligned}$$

one can obtain via (9) and (10) that

$$\begin{aligned} \mathop {\lim }\limits _{t \rightarrow + \infty } \left( {{\zeta _i}(t) - \frac{1}{{\sqrt{M} }}{{{\tilde{\zeta }} }_1}(t)} \right) = {\textbf{0}}\quad (i = 1,2, \ldots ,M) \end{aligned}$$

if and only if ${\lim _{t \rightarrow + \infty }}{{\tilde{\zeta }} _i}(t) = 0\,\,(i = 2,3, \ldots ,M)$. Due to ${\zeta _i}(t) = {x_i}(t) - {\xi _i}(t)\,\,(i = 1,2, \ldots ,M),$ one has

11 $$\begin{aligned} \mathop {\lim }\limits _{t \rightarrow + \infty } \left( {{x_i}(t) - {\xi _i}(t) - \frac{1}{{\sqrt{M} }}{{{\tilde{\zeta }} }_1}(t)} \right) = {\textbf{0}}\quad (i = 1,2, \ldots ,M), \end{aligned}$$

which guarantees that ${\lim _{t \rightarrow + \infty }}\left( {{x_k}(t) - {x_i}(t) - {\xi _k}(t) + {\xi _i}(t)} \right) = {\textbf{0}}$ $\left( {\forall i,k \in \left\{ {1,2, \ldots ,M} \right\} } \right)$ and ${{\tilde{\zeta }} _1}(t)$ can be used to depict the motion trajectory of the formation center. In other words, ${{\tilde{\zeta }} _w}(t)$ and ${{\tilde{\zeta }} _r}(t)$ describe the formation motion as a whole and the relative motions among agents, respectively. Thus, the above formation problem is transformed into the asymptotic stability one.

Furthermore, we give a design approach of the gain matrix such that the formation of interconnected system (1) with formation protocol (P1) can be achieved. Let ${P^T} = P > 0$, then consider a common Lyapunov functional candidate as follows:

12 $$\begin{aligned} V(t) = \sum \limits _{i = 2}^M {{\tilde{\zeta }} _i^T(t){P^{ - 1}}{{{\tilde{\zeta }} }_i}(t)} . \end{aligned}$$

Then

13 $$\begin{aligned} \dot{V}(t) = \sum \limits _{i = 2}^M {\left( {{V_{i1}}(t) + {V_{i2}}(t) - {V_{i3}}(t)} \right) } , \end{aligned}$$

where

$$\begin{aligned} {V_{i1}}(t)&= {\tilde{\zeta }} _i^T(t){P^{ - 1}}\left( {\left( {A - {\lambda _i}BK} \right) P + P{{\left( {A - {\lambda _i}BK} \right) }^T}} \right) {P^{ - 1}}{{\tilde{\zeta }} _i}(t),\\ {V_{i2}}(t)&= 2{\tilde{\zeta }} _i^T(t){P^{ - 1}}\left( {e_i^T{U^T} \otimes A} \right) \xi (t), \\ {V_{i3}}(t)&= 2{\tilde{\zeta }} _i^T(t){P^{ - 1}}\left( {e_i^T{U^T} \otimes {I_d}} \right) \dot{\xi }(t). \end{aligned}$$

For $i = 2,3, \ldots ,M,$ based on the mathematics regulation of the Kronecker product, there exists

$$\begin{aligned} e_i^T{U^T} \otimes A = \left( {e_i^T{U^T} \otimes {I_d}} \right) \left( {{I_M} \otimes A} \right) . \end{aligned}$$

Thus,

14 $$\begin{aligned} {V_{i2}}(t) - {V_{i3}}(t) = 2{\tilde{\zeta }} _i^T(t){P^{ - 1}}\left( {e_i^T{U^T} \otimes {I_d}} \right) \left( {\left( {{I_M} \otimes A} \right) \xi (t) - \dot{\xi }(t)} \right) . \end{aligned}$$

If $$A{\xi _i}(t) = {\dot{\xi }_i}(t)$$ $$\left( {i = 1,2, \ldots ,M} \right)$$, then

$$\begin{aligned} \left( {{I_M} \otimes A} \right) \xi (t) = \dot{\xi }(t), \end{aligned}$$

which means via (14) that ${V_{i2}}(t) - {V_{i3}}(t) = 0$. If $K = \lambda _2^{ - 1}{B^T}{P^{ - 1}}$, then one can derive by (13) that

15 $$\begin{aligned} \dot{V}(t) = \sum \limits _{i = 2}^M {{\tilde{\zeta }} _i^T(t){P^{ - 1}}\left( {AP + P{A^T} - 2\lambda _2^{ - 1}{\lambda _i}B{B^T}} \right) {P^{ - 1}}{{{\tilde{\zeta }} }_i}(t)} . \end{aligned}$$

Since $\lambda _2^{ - 1}{\lambda _i} \geqslant 1$ $\left( {i = 2,3, \ldots ,M} \right)$, the linear matrix inequality $AP + P{A^T} - 2B{B^T} < 0$ can guarantee that the $M - 1$ linear matrix inequalities $AP + P{A^T} - 2\lambda _2^{ - 1}{\lambda _i}B{B^T} < 0\,\,\left( {i = 2,3, \ldots ,M} \right) .$ From (15), for any nonzero ${{\tilde{\zeta }} _i}(t)\,\,\left( {i = 2,3, \ldots ,M} \right)$ $\dot{V}(t) < 0$ if $R{A^T} + AR - 2B{B^T} < 0$, and $\dot{V}(t) \equiv 0$ if and only if ${{\tilde{\zeta }} _i}(t) \equiv {\textbf{0}}\,\,(i = 2,3, \ldots ,M)$; that is, subsystems (5) can be simultaneously stabilized by the gain matrix $K = \lambda _2^{ - 1}{B^T}{P^{ - 1}}$. In this case, interconnected system (1) with formation protocol (P1) achieves formation.

In addition,, the impacts of the energy restriction on formation achievement are dealt with. By formation protocol (P1), one has

$$\begin{aligned} E = \int _0^{ + \infty } {{\zeta ^T}(t)({L^2} \otimes {K^T}WK)\zeta (t){\text {d}}t}. \end{aligned}$$

Because *U* is orthogonal and $\left( {{U^T} \otimes {I_d}} \right) \zeta (t) = {\left[ {{\tilde{\zeta }} _1^T(t),{\tilde{\zeta }} _2^T(t), \ldots ,{\tilde{\zeta }} _M^T(t)} \right] ^T}$, one can show that

$$\begin{aligned} E = \sum \limits _{i = 2}^M {\int _0^{ + \infty } {\lambda _i^2{\tilde{\zeta }} _i^T(t){K^T}WK{{{\tilde{\zeta }} }_i}(t){\text {d}}t} }. \end{aligned}$$

Let $K = \lambda _2^{ - 1}{B^T}{P^{ - 1}}$, then one has

16 $$\begin{aligned} E = \sum \limits _{i = 2}^M {\int _0^{ + \infty } {{{\left( {\lambda _2^{ - 1}{\lambda _i}} \right) }^2}\left( {{\tilde{\zeta }} _i^T(t){P^{ - 1}}} \right) BW{B^T}\left( {{P^{ - 1}}{{{\tilde{\zeta }} }_i}(t)} \right) {\text {d}}t} } . \end{aligned}$$

Since ${\lim _{t \rightarrow + \infty }}V(t) = 0$ and ${V_{i2}}(t) - {V_{i3}}(t) = 0\,\,\left( {i = 2,3, \ldots ,M} \right)$, one can derive by (16) that

17 $$\begin{aligned} E = \sum \limits _{i = 2}^M {\int _0^{ + \infty } {\left( {{V_{i1}}(t) + {{\left( {\lambda _2^{ - 1}{\lambda _i}} \right) }^2}\left( {{\tilde{\zeta }} _i^T(t){P^{ - 1}}} \right) BW{B^T}\left( {{P^{ - 1}}{{{\tilde{\zeta }} }_i}(t)} \right) } \right) {\text {d}}t + V(0)} } . \end{aligned}$$

By Schur lemma in^30^, if ${\Omega _1} < 0,$ then one can find that for $\forall i \in \left\{ {2,3, \ldots ,M} \right\} ,$ the following matrix inequalities hold:

$$\begin{aligned} AP + P{A^T} - 2\lambda _2^{ - 1}{\lambda _i}B{B^T} + {\left( {\lambda _2^{ - 1}{\lambda _i}} \right) ^2}BW{B^T} < 0. \end{aligned}$$

From (17), it can be found that

18 $$\begin{aligned} E \leqslant \sum \limits _{i = 2}^M {{\tilde{\zeta }} _i^T(0){P^{ - 1}}{{{\tilde{\zeta }} }_i}(0)} . \end{aligned}$$

According to

$$\begin{aligned} \left( {{U^T} \otimes {I_d}} \right) \zeta (0) = {\left[ {{\tilde{\zeta }} _1^T(0),{\tilde{\zeta }} _2^T(0), \ldots ,{\tilde{\zeta }} _M^T(0)} \right] ^T}, \end{aligned}$$

it can be deduced that

$$\begin{aligned} {\left[ {{\tilde{\zeta }} _2^T(0),{\tilde{\zeta }} _3^T(0), \ldots ,{\tilde{\zeta }} _M^T(0)} \right] ^T} = \left( {\left[ {{\textbf{0}},{I_{M - 1}}} \right] {U^T} \otimes {I_d}} \right) \zeta (0). \end{aligned}$$

Hence, one can show that

19 $$\begin{aligned} \sum \limits _{i = 2}^M {{\tilde{\zeta }} _i^T(0){{{\tilde{\zeta }} }_i}(0)} = {\zeta ^T}(0)\left( {U{{\left[ {{\textbf{0}},{I_{M - 1}}} \right] }^T}\left[ {{\textbf{0}},{I_{M - 1}}} \right] {U^T} \otimes {I_d}} \right) \zeta (0). \end{aligned}$$

Due to $U{e_1} = {{\textbf{1}} / {\sqrt{M} }}$, one can set that $U = \left[ {{{\textbf{1}} /{\sqrt{M} }},{\tilde{U}}} \right]$ with ${\tilde{U}} \in {\mathbb {R}^{M \times (M - 1)}}$. Since $U{U^T} = {I_M},$ one can derive that

20 $$\begin{aligned} {\tilde{U}}{{\tilde{U}}^T} = {I_M} - \frac{1}{M}{\textbf{1}}{{\textbf{1}}^T}. \end{aligned}$$

By (19) and (20), one has

21 $$\begin{aligned} \sum \limits _{i = 2}^M {{\tilde{\zeta }} _i^T(0){{{\tilde{\zeta }} }_i}(0)} = {\zeta ^T}(0)\left( {\left( {{I_M} - \frac{1}{M}{\textbf{1}}{{\textbf{1}}^T}} \right) \otimes {I_d}} \right) \zeta (0). \end{aligned}$$

Since the relative motions among agents are described by ${{\tilde{\zeta }} _r}(t)$ and it is supposed that initial conditions are disagreement, it can be shown that

22 $$\begin{aligned} \sum \limits _{i = 2}^M {{\tilde{\zeta }} _i^T(0){{{\tilde{\zeta }} }_i}(0)} > 0. \end{aligned}$$

There exists a scalar $\eta$ such that

23 $$\begin{aligned} {E_\eta } = {\zeta ^T}(0)\left( {\left( {{I_M} - \frac{1}{M}{\textbf{1}}{{\textbf{1}}^T}} \right) \otimes \eta {I_d}} \right) \zeta (0). \end{aligned}$$

where the positive scalar $\eta$ is called the optimal factor. By introducing the optimal factor $\eta$, the explicit expression (23) of the total energy restriction is constructed. Due to (21)–(23), it can be found that the total energy restriction can be minimized by minimizing $\eta$. Especially, by (18), one can also show that

24 $$\begin{aligned} E \leqslant {\zeta ^T}(0)\left( {\left( {{I_M} - \frac{1}{M}{\textbf{1}}{{\textbf{1}}^T}} \right) \otimes {P^{ - 1}}} \right) \zeta (0). \end{aligned}$$

By (23) and (24), the energy restriction minimization can be achieved by minimizing the positive scalar $\eta$ by ${P^{ - 1}} < \eta {I_d}$, which is equivalent to ${I_d} - \eta P < 0$; that is, ${\Omega _2} < 0.$ The proof of Theorem 1 is completed. $\square$

In Theorem 1, the conclusion depends on the maximum and minimum nonzero eigenvalues of the Laplacian matrix, which are introduced by the energy restriction and can be removed when the energy restriction term is removed. It should be pointed out that these two eigenvalues are used to constrain the linear matrix inequality condition and their estimated values are also valid. Furthermore, the detailed estimated approaches can be found in^31^ and^32^, respectively, and they have the lower computation complexity compared with solving their precise values.

Moreover, based on the proofs of Theorem 1, for any given gain matrix *K*, the following conclusion can be derived directly, which presents a sufficient condition of minimum-energy-restriction formation analysis for interconnected system (1) with formation protocol (P1), where the convex property of the linear matrix inequality is applied to decrease the computation complexity.

### Corollary 1

For any given *K*, interconnected system (1) with formation protocol (P1) achieves minimum-energy-restriction formation if $A{\xi _i}(t) = {\dot{\xi }_i}(t)$ $\left( {i = 1,2, \ldots ,M} \right)$ and there exists ${{\tilde{P}}^T} = {\tilde{P}} > 0$ such that the following minimization problem has an optimal solution $\eta$:

$$\begin{aligned} \hbox {min } & \quad \eta \\ \hbox {s. t.} & \quad {\tilde{\Omega }} _1^i = \left[ {\begin{array}{*{20}{c}} {{A^T}{\tilde{P}} + {\tilde{P}}A - {\lambda _i}{K^T}{B^T}{\tilde{P}} - {\lambda _i}{\tilde{P}}BK}&{{\lambda _i}{K^T}W} \\ * &{ - W} \end{array}} \right]< 0{{ }}(i = 2,M),\\ & \quad {{\tilde{\Omega }} _2} = {\tilde{P}} - \eta {I_d} < 0. \end{aligned}$$

When minimum-energy-restriction formation is achieved, the agents of interconnected system (1) move in a specific formation. The formation motion trajectory as a whole can be depicted by ${{\tilde{\zeta }} _w}(t)$ as shown in the proofs of Theorem 1. Due to ${\tilde{\zeta }} (t) = \left( {{U^T} \otimes {I_d}} \right) \zeta (t)$, one can show that ${{\tilde{\zeta }} _1}(0) = \left( {e_1^T{U^T} \otimes {I_d}} \right) \zeta (0) = \sum \nolimits _{i = 1}^M {{{{\zeta _i}(0)} / {\sqrt{M} }}} .$ By (4), (9) and (11), one can obtain the following corollary, which determines the formation whole motion trajectory of interconnected system (1) with formation protocol (P1).

### Corollary 2

If interconnected system (1) with formation protocol (P1) achieves minimum-energy-restriction formation, then the formation whole motion trajectory ${\zeta _w}(t)$ satisfies that

$$\begin{aligned} \mathop {\lim }\limits _{t \rightarrow + \infty } \left( {{\zeta _w}(t) - {\zeta _x}(t) - {\zeta _\xi }(t)} \right) = 0, \end{aligned}$$

where

$$\begin{aligned} {\zeta _x}(t) = {e^{At}}\left( {\frac{1}{M}\sum \limits _{i = 1}^M {{x_i}(0)} } \right) , \\ {\zeta _\xi }(t) = - {e^{At}}\left( {\frac{1}{M}\sum \limits _{i = 1}^M {{\xi _i}(0)} } \right) . \end{aligned}$$

### Remark 2

In order to establish the relationship between the control gain matrix and the energy restriction in Theorem 1, a positive scalar and the matrix variable of the Lyapunov function are introduced. The positive scalar is used to transform the energy sum of all agents into an explicit expression, and the matrix variable is applied to obtain the upper bound of the whole energy. In this case, the gain matrix can be regulated to make the energy restriction minimum and the optimization problem can be solved by the optimization approach of the generalized eigenvalue as shown in^30^. It should be noted that the positive scalar is minimum in the sense of the linear matrix inequality, but it is not a globally optimal solution. Furthermore, the formation feasible condition $A{\xi _i}(t) = {\dot{\xi }_i}(t)$ $\left( {i = 1,2, \ldots ,M} \right)$ is proposed, which is necessary and sufficient as shown in our previous work^33^. Especially, the formation feasible condition shows that the physical structure of each agent is an important factor for formation feasibility. Moreover, Corollary 2 determines the formation motion trajectory of an interconnected system as a whole, where the effects of the initial states and formation control vectors are given, respectively. It can be found that the formation whole motion trajectory is related to the average of initial states, which is essentially determined by the fact that the information topology is undirected and connected.

## Minimum-energy-restriction formation with interaction silence

In this section, a formation protocol with the interaction silence is proposed, where interconnected channels among all agents are removed during the interaction silence subsegment. Then, by introducing the interaction silence rate and two inhibition parameters, the divergent property during the interaction silence subsegment is handled and sufficient conditions for minimum-energy-restriction formation are discussed.

There may exist the channel jamming or active anti-jamming, so the non-periodic interaction silence may appear in interconnected channels among agents. In other words, all information transmissions are removed in a passive or active manner. The whole time interval can be divided into infinite and non-overlapping time segments $\left[ {{t_j},{t_{j + 1}}} \right) \,\,\left( {\forall j \in {{\bar{\mathbb {Z}}}^ - }} \right)$ with ${t_0} = 0$ and the union of these segments is the whole time interval. Especially, this division is non-periodic; that is, time segments may own different lengths. Let ${t_{\max }}$ and ${t_{\min }}$ denote the maximum and minimum time segments, respectively; that is, ${t_{\max }} = \max \left\{ {{t_{j + 1}} - {t_j}:\forall j \in {{\bar{\mathbb {Z}}}^ - }} \right\}$ and ${t_{\min }} = \min \left\{ {{t_{j + 1}} - {t_j}:\forall j \in {{\bar{\mathbb {Z}}}^ - }} \right\}$. For $\forall j \in {\bar{\mathbb {Z}}^ - },$ the associated segment is separated into two subsegments: the non-silence subsegment $\left[ {{t_j},{{{\tilde{t}}}_j}} \right)$ and the silence subsegment $\left[ {{{{\tilde{t}}}_j},{t_{j + 1}}} \right)$, whose lengths can be unequal. It should be pointed out that the cooperative information is conveyed among neighboring agents during the non-silence subsegment $\left[ {{t_j},{{{\tilde{t}}}_j}} \right)$, but no cooperative information is conveyed among agents during the silence subsegment $\left[ {{{{\tilde{t}}}_j},{t_{j + 1}}} \right)$. The interaction silence rate of the time segment $\left[ {{t_j},{t_{j + 1}}} \right) \,\,\left( {\forall j \in {{\bar{ \mathbb {Z}}}^ - }} \right)$ is defined as ${\varpi _j} = {{\left( {{t_{j + 1}} - {{{\tilde{t}}}_j}} \right) } /{\left( {{t_{j + 1}} - {t_j}} \right) }}$ and an upper bound of silence rates of all time segments is denoted as ${\tilde{\varpi }}$; that is, $0 \leqslant {\varpi _j} \leqslant {\tilde{\varpi }} < 1$ $\left( {\forall j \in {{\bar{\mathbb {Z}}}^ - }} \right)$.

By synthesizing the cooperative states and the formation control vectors of neighboring agents, a formation protocol with the energy restriction and the non-periodic interaction silence is proposed as follows:

P2 $$\begin{aligned} \left\{ \begin{array}{ll} {u_i}(t) = K\sum \limits _{k \in {\mathcal {N}_i}} {{w_{ik}}\left( {{x_k}(t) - {\xi _k}(t) - {x_i}(t) + {\xi _i}(t)} \right) } {{, }}t \in \left[ {{t_j},{{{\tilde{t}}}_j}} \right) , \\ {u_i}(t) = {\textbf{0}},{{ }}t \in \left[ {{{{\tilde{t}}}_j},{t_{j + 1}}} \right) , \\ E = \sum \limits _{i = 1}^M {\int _0^{ + \infty } {u_i^T(t)W{u_i}(t){\text {d}}t} } , \\ \end{array} \right. \end{aligned}$$

where $i = 1,2, \ldots ,M,$ $\forall j \in {\bar{\mathbb {Z}}^ - },$ and the other notations have the same meanings as formation protocol (P1). During the silence subsegment $\left[ {{{{\tilde{t}}}_j},{t_{j + 1}}} \right)$ $\left( {\forall j \in {{\bar{\mathbb {Z}}}^ - }} \right)$, the control inputs of all agents are equal to zero since no cooperative information is transmitted among agents. Furthermore, it can be found that the cooperative state of each agent may diverge away during the silence subsegment $\left[ {{{{\tilde{t}}}_j},{t_{j + 1}}} \right)$ $\left( {\forall j \in {{\bar{\mathbb {Z}}}^ - }} \right)$ if the system matrix *A* is Lyapunov unstable. Intuitionally speaking, the formation achievement of interconnected system (1) with formation protocol (P2) can be guaranteed if the convergent quantity during the non-silence subsegment is larger than the divergent quantity during the silence subsegment.

During the silence subsegment $\left[ {{{{\tilde{t}}}_j},{t_{j + 1}}} \right)$ $\left( {\forall j \in {{\bar{\mathbb {Z}}}^ - }} \right)$, the associated Laplacian matrix of interconnected system (1) is a zero matrix. In this case, by the similar analysis to the case without the interaction silence, the whole dynamics of interconnected system (1) with formation protocol (P2) can be transformed into

25 $$\begin{aligned}&{\dot{{\tilde{\zeta }}} _1}(t) = A{{\tilde{\zeta }} _1}(t) + \left( {e_1^T{U^T} \otimes A} \right) \xi (t) - \left( {e_1^T{U^T} \otimes {I_d}} \right) \dot{\xi }(t),\,\,t \in \left[ {{t_j},{t_{j + 1}}} \right) {\text {,}} \end{aligned}$$

26 $$\begin{aligned}&\left\{ \begin{array}{ll} {{\dot{{\tilde{\zeta }}} }_i}(t) = \left( {A - {\lambda _i}BK} \right) {{{\tilde{\zeta }} }_i}(t) + \left( {e_i^T{U^T} \otimes A} \right) \xi (t) - \left( {e_i^T{U^T} \otimes {I_d}} \right) \dot{\xi }(t),\,\,t \in \left[ {{t_j},{{{\tilde{t}}}_j}} \right) {\text {,}} \\ {{\dot{{\tilde{\zeta }}} }_i}(t) = A{{{\tilde{\zeta }} }_i}(t) + \left( {e_i^T{U^T} \otimes A} \right) \xi (t) - \left( {e_i^T{U^T} \otimes {I_d}} \right) \dot{\xi }(t),t \in \left[ {{{{\tilde{t}}}_j},{t_{j + 1}}} \right) , \\ \end{array} \right. \end{aligned}$$

where $i = 2,3, \ldots ,M$ and all notations are identical to the case without the interaction silence. However, it can be found that subsystems (26) are divided into two parts, which are related to the non-silence subsegment and the silence subsegment, respectively. It can be shown that subsystems (25) and (26) characterize the whole motion of the formation and the relative motion among agents, respectively. Moreover, the critical challenge is to handle the impacts of the interaction silence. In other words, it is required to find an approach to simultaneously stabilize subsystems (26) with the interaction silence. A sufficient condition for minimum-energy-restriction formation design with the interaction silence is presented by the following theorem.

### Theorem 2

Interconnected system (1) is minimum-energy-restriction formation achievable by formation protocol (P2) with $K = \lambda _2^{ - 1}{B^T}{{\bar{P}}^{ - 1}}$ if $A{\xi _i}(t) = {\dot{\xi }_i}(t)$ $\left( {i = 1,2, \ldots ,M} \right)$ and there exists $\alpha > 0,$ $\beta > 0$ and ${{\bar{P}}^T} = {\bar{P}} > 0$ such that $\beta (1 - {\tilde{\varpi }} ) > \alpha {\tilde{\varpi }} {e^{\alpha {\tilde{\varpi }} {t_{\max }}}}$ and the following minimization problem has an optimal solution $\eta$:

$$\begin{aligned} \hbox {min} & \quad \eta \\ \hbox {s. t.} & \quad {{\bar{\Omega }} _1} = A{\bar{P}} + {\bar{P}}{A^T} - \alpha {\bar{P}}< 0, \\ & \quad {{\bar{\Omega }} _2} = \left[ {\begin{array}{*{20}{c}} {{\bar{P}}{A^T} + A{\bar{P}} - 2B{B^T} + \beta {\bar{P}}}&{\lambda _2^{ - 1}{\lambda _M}BW} \\ * &{ - W} \end{array}} \right]< 0,\\ & \quad {{\bar{\Omega }} _3} = {I_d} - \eta {\bar{P}} < 0. \end{aligned}$$

### Proof

If $A{\xi _i}(t) = {\dot{\xi }_i}(t)$ $\left( {i = 1,2, \ldots ,M} \right)$, then from (26) one has

27 $$\begin{aligned} \left\{ \begin{array}{ll} {{\dot{{\tilde{\zeta }}} }_i}(t) = \left( {A - {\lambda _i}BK} \right) {{{\tilde{\zeta }} }_i}(t),\,\,t \in \left[ {{t_j},{{{\tilde{t}}}_j}} \right) {\text {,}} \\ {{\dot{{\tilde{\zeta }}} }_i}(t) = A{{{\tilde{\zeta }} }_i}(t),\,\,t \in \left[ {{{{\tilde{t}}}_j},{t_{j + 1}}} \right) , \\ \end{array} \right. \end{aligned}$$

where $i = 2,3, \ldots ,M.$ Construct the following Lyapunov functional candidates:

28 $$\begin{aligned} {{\bar{V}}_i}(t) = {\tilde{\zeta }} _i^T(t){{\bar{P}}^{ - 1}}{{\tilde{\zeta }} _i}(t)\,\,\left( {\forall i \in \left\{ {2,3, \ldots ,M} \right\} } \right) , \end{aligned}$$

where ${{\bar{P}}^T} = {\bar{P}} > 0$. During the silence subsegment $\left[ {{{{\tilde{t}}}_j},{t_{j + 1}}} \right)$ with $\forall j \in {\bar{\mathbb {Z}}^ - }$, ${\dot{{\bar{V}}}_i}(t)$ can be solved as

29 $$\begin{aligned} {\dot{ {\bar{V}}}_i}(t) = {\tilde{\zeta }} _i^T(t){{\bar{P}}^{ - 1}}\left( {A{\bar{P}} + {\bar{P}}{A^T}} \right) {{\bar{P}}^{ - 1}}{{\tilde{\zeta }} _i}(t). \end{aligned}$$

In this case, when $A{\bar{P}} + {\bar{P}}{A^T} - \alpha {\bar{P}} < 0$ with $\alpha > 0$, then

30 $$\begin{aligned} {\dot{{\bar{V}}}_i}(t) < \alpha {{\bar{V}}_i}(t). \end{aligned}$$

During the non-silence subsegment $t \in \left[ {{t_j},{{{\tilde{t}}}_j}} \right)$ with $\forall j \in {\bar{\mathbb {Z}}^ - }$, one has

$$\begin{aligned} {\dot{{\bar{V}}}_i}(t) = {\tilde{\zeta }} _i^T(t)\left( {{A^T}{{{\bar{P}}}^{ - 1}} + {{{\bar{P}}}^{ - 1}}A - {\lambda _i}{K^T}{B^T}{{{\bar{P}}}^{ - 1}} - {\lambda _i}{{{\bar{P}}}^{ - 1}}BK} \right) {{\tilde{\zeta }} _i}(t). \end{aligned}$$

In this case, let $K = \lambda _2^{ - 1}{B^T}{{\bar{P}}^{ - 1}}$, then one can show that

$$\begin{aligned} {\dot{{\bar{V}}}_i}(t) = {\tilde{\zeta }} _i^T(t){{\bar{P}}^{ - 1}}\left( {A{\bar{P}} + {\bar{P}}{A^T} - 2{\lambda _i}\lambda _2^{ - 1}B{B^T}} \right) {{\bar{P}}^{ - 1}}{{\tilde{\zeta }} _i}(t). \end{aligned}$$

Due to ${\lambda _i}\lambda _2^{ - 1} \geqslant 1\,\,\left( {\forall i \in \left\{ {2,3, \ldots ,M} \right\} } \right)$, one has

31 $$\begin{aligned} {\dot{{\bar{V}}}_i}(t) \leqslant {\tilde{\zeta }} _i^T(t){{\bar{P}}^{ - 1}}\left( {A{\bar{P}} + {\bar{P}}{A^T} - 2B{B^T}} \right) {{\bar{P}}^{ - 1}}{{\tilde{\zeta }} _i}(t). \end{aligned}$$

If $A{\bar{P}} + {\bar{P}}{A^T} - 2B{B^T} + \beta {\bar{P}} < 0$ with $\beta > 0$, by (28) and (31) one has

32 $$\begin{aligned} {\dot{{\bar{V}}}_i}(t) < - \beta {{\bar{V}}_i}(t). \end{aligned}$$

For $j = 0$, it can be derived by (30) and (32) that

33 $$\begin{aligned}&{{\bar{V}}_i}({t_1}) < {e^{\alpha ({t_1} - {{{\tilde{t}}}_0})}}{{\bar{V}}_i}({{\tilde{t}}_0}), \end{aligned}$$

34 $$\begin{aligned}&{{\bar{V}}_i}({{\tilde{t}}_0}) < {e^{ - \beta \left( {{{{\tilde{t}}}_0} - {t_0}} \right) }}{{\bar{V}}_i}(0). \end{aligned}$$

Let ${{\hat{\varpi }} _0} = ((\beta + \alpha ){\varpi _0} - \beta )({t_1} - {t_0})$, then by (33) and (34), one can show that

35 $$\begin{aligned} {{\bar{V}}_i}({t_1}) < {e^{{{{\hat{\varpi }} }_0}}}{{\bar{V}}_i}(0). \end{aligned}$$

By $\alpha > 0$ and ${\tilde{\varpi }} > 0$, it can be found that ${e^{\alpha {\tilde{\varpi }} {t_{\max }}}} > 1$. Hence, one can show that $\beta (1 - {\tilde{\varpi }} ) > \alpha {\tilde{\varpi }}$ if $\beta (1 - {\tilde{\varpi }} ) > \alpha {\tilde{\varpi }} {e^{\alpha {\tilde{\varpi }} {t_{\max }}}}$. Thus, one can obtain that ${{\hat{\varpi }} _0} < 0$ by (35), and ${{\bar{V}}_i}(t)\,\,\left( {t \in \left[ {{t_0},{t_1}} \right) } \right)$ decreases exponentially. Furthermore, one also can deduce that ${{\hat{\varpi }} _j} = ((\beta + \alpha ){\varpi _j} - \beta )({t_{j + 1}} - {t_j}) < 0$ with $\forall j \in {\bar{\mathbb {Z}}^ - }.$ For any integer $\varsigma > 0$, one can show that

$$\begin{aligned} {{\bar{V}}_i}({t_\varsigma }) < {e^{\sum \limits _{j = 0}^{\varsigma - 1} {{{{\hat{\varpi }} }_j}} }}{{\bar{V}}_i}(0). \end{aligned}$$

It can be obtained that the convergent quantity of the Lyapunov function ${{\bar{V}}_i}(t)$ is associated with the silence rate ${\varpi _j}$ in each time subsegment $\left[ {{t_j},{t_{j + 1}}} \right) \mathrm{{ }}\left( \forall j \in {\bar{\mathbb {Z}}^ - } \right)$, and the influence of the silence rate on the convergent quantity is piecewise accumulated. When the silence rate is larger than a certain value, the interconnected system may diverge away and cannot achieve the distributed formation. For any $t > 0$, one can find an integer $\upsilon > 0$ such that ${t_\upsilon }< t < {t_{\upsilon + 1}}$. One has

36 $$\begin{aligned} {{\bar{V}}_i}(t)< {e^{\alpha {t_{\max }}}}{{\bar{V}}_i}({t_\upsilon }) < {e^{\alpha {t_{\max }}}}{e^{\sum \limits _{j = 0}^{\upsilon - 1} {{{{\hat{\varpi }} }_j}} }}{{\bar{V}}_i}(0). \end{aligned}$$

According to

$$\begin{aligned} & t \leqslant \upsilon {t_{\max }}, \\ & \sum \limits _{j = 0}^{\upsilon - 1} {{{{\hat{\varpi }} }_j}} \leqslant \upsilon ((\beta + \alpha ){\tilde{\varpi }} - \beta ){t_{\min }}, \end{aligned}$$

one has

$$\begin{aligned} \sum \limits _{j = 0}^{\upsilon - 1} {{{{\hat{\varpi }} }_j}} \leqslant \frac{{((\beta + \alpha ){\tilde{\varpi }} - \beta ){t_{\min }}}}{{{t_{\max }}}}t. \end{aligned}$$

From (36), one has

37 $$\begin{aligned} {{\bar{V}}_i}(t) < {e^{\alpha {t_{\max }}}}{e^{\frac{{((\beta + \alpha ){\tilde{\varpi }} - \beta ){t_{\min }}}}{{{t_{\max }}}}t}}{{\bar{V}}_i}(0). \end{aligned}$$

Since $(\beta + \alpha ){\tilde{\varpi }} - \beta < 0$, it can be found that ${\lim _{t \rightarrow {{ + }}\infty }}{{\bar{V}}_i}(t) = 0$. Due to ${{\bar{P}}^T} = {\bar{P}} > 0$, one can get that ${\lim _{t \rightarrow + \infty }}{{\tilde{\zeta }} _i}(t) = {\textbf{0}}$ $\left( {i = 2,3, \ldots ,M} \right)$; that is, subsystems (27) are simultaneously stabilized by $K = \lambda _2^{ - 1}{B^T}{{\bar{P}}^{ - 1}}$, which means that interconnected system (1) is formation achievable by formation protocol (P2) with $K = \lambda _2^{ - 1}{B^T}{{\bar{P}}^{ - 1}}$.

Furthermore, we determine the effects of the interaction silence on the energy restriction. Since the control input is zero in $t \in \left[ {{{{\tilde{t}}}_j},{t_{j + 1}}} \right)$ with $\forall j \in {\bar{\mathbb {Z}}^ - }$, one has

$$\begin{aligned} \int _0^{ + \infty } {u_i^T(t)W{u_i}(t){\text {d}}t} = \sum \limits _{j = 0}^{ + \infty } {\int _{{t_j}}^{{{{\tilde{t}}}_j}} {u_i^T(t)W{u_i}(t){\text {d}}t} }. \end{aligned}$$

According to formation protocol (P2), one has

$$\begin{aligned} E = \sum \limits _{j = 0}^{ + \infty } {\int _{{t_j}}^{{{{\tilde{t}}}_j}} {{\zeta ^T}(t)({L^2} \otimes {K^T}WK)\zeta (t){\text {d}}t} }. \end{aligned}$$

By the orthogonal transformation matrix *U* and $K = \lambda _2^{ - 1}{B^T}{{\bar{P}}^{ - 1}}$, one can show that

38 $$\begin{aligned} E = \sum \limits _{i = 2}^M {\sum \limits _{j = 0}^{ + \infty } {\int _{{t_j}}^{{{{\tilde{t}}}_j}} {{{\left( {\lambda _2^{ - 1}{\lambda _i}} \right) }^2}\left( {{\tilde{\zeta }} _i^T(t){{{\bar{P}}}^{ - 1}}} \right) BW{B^T}\left( {{{{\bar{P}}}^{ - 1}}{{{\tilde{\zeta }} }_i}(t)} \right) {\text {d}}t} } } . \end{aligned}$$

Moreover, one has

39 $$\begin{aligned}&{\dot{{\bar{V}}}_i}(t) - \alpha {{\bar{V}}_i}(t) = {\tilde{\zeta }} _i^T(t){{\bar{P}}^{ - 1}}\left( {A{\bar{P}} + {\bar{P}}{A^T} - \alpha {\bar{P}}} \right) {{\bar{P}}^{ - 1}}{{\tilde{\zeta }} _i}(t),t \in \left[ {{{{\tilde{t}}}_j},{t_{j + 1}}} \right) , \end{aligned}$$

40 $$\begin{aligned}&{\dot{{\bar{V}}}_i}(t) + \beta {{\bar{V}}_i}(t) \leqslant {\tilde{\zeta }} _i^T(t){{\bar{P}}^{ - 1}}\left( {A{\bar{P}} + {\bar{P}}{A^T} - 2B{B^T} + \beta {\bar{P}}} \right) {{\bar{P}}^{ - 1}}{{\tilde{\zeta }} _i}(t),t \in \left[ {{t_j},{{{\tilde{t}}}_j}} \right) , \end{aligned}$$

where $i = 2,3, \ldots ,M$ and $\forall j \in {\bar{\mathbb {Z}}^ - }.$ From (38) to (40), it can be derived that

41 $$\begin{aligned} \begin{aligned} E&\leqslant \sum \limits _{i = 2}^M {\sum \limits _{j = 0}^{ + \infty } {\int _{{t_j}}^{{{{\tilde{t}}}_j}} {{\tilde{\zeta }} _i^T(t){{{\bar{P}}}^{ - 1}}\left( {A{\bar{P}} + {\bar{P}}{A^T} - 2B{B^T} + \beta {\bar{P}} + {{\left( {\lambda _2^{ - 1}{\lambda _M}} \right) }^2}BW{B^T}} \right) {{{\bar{P}}}^{ - 1}}{{{\tilde{\zeta }} }_i}(t){\text {d}}t} } } \\&\quad + \sum \limits _{i = 2}^M {\sum \limits _{j = 0}^{ + \infty } {\int _{{{{\tilde{t}}}_j}}^{{t_{j + 1}}} {{\tilde{\zeta }} _i^T(t){{{\bar{P}}}^{ - 1}}\left( {A{\bar{P}} + {\bar{P}}{A^T} - \alpha {\bar{P}}} \right) {{{\bar{P}}}^{ - 1}}{{{\tilde{\zeta }} }_i}(t){\text {d}}t} } } \\&\quad - \sum \limits _{i = 2}^M {\int _0^{ + \infty } {{{\dot{{\bar{V}}}}_i}(t){\text {d}}t} } - \sum \limits _{i = 2}^M {\sum \limits _{j = 0}^{ + \infty } {\left( {\int _{{t_j}}^{{{{\tilde{t}}}_j}} {\beta {{{\bar{V}}}_i}(t){\text {d}}t} - \int _{{{{\tilde{t}}}_j}}^{{t_{j + 1}}} {\alpha {{{\bar{V}}}_i}(t){\text {d}}t} } \right) } } . \\ \end{aligned} \end{aligned}$$

From Schur lemma, if ${{\bar{\Omega }} _i} < 0\,\,\left( {i = 1,2} \right)$, then it can be obtained via (41) that

42 $$\begin{aligned} E \leqslant \sum \limits _{i = 2}^M {{{{\bar{V}}}_i}(0)} + \sum \limits _{i = 2}^M {\sum \limits _{j = 0}^{ + \infty } {\left( {\alpha \left( {{t_{j + 1}} - {{{\tilde{t}}}_j}} \right) {{{\bar{V}}}_i}({t_{j + 1}}) - \beta \left( {{{{\tilde{t}}}_j} - {t_j}} \right) {{{\bar{V}}}_i}({{{\tilde{t}}}_j})} \right) } } . \end{aligned}$$

By ${{\bar{V}}_i}({t_{j + 1}}) < {e^{\alpha ({t_{j + 1}} - {{{\tilde{t}}}_j})}}{{\bar{V}}_i}({{\tilde{t}}_j}),$ one has

43 $$\begin{aligned} \alpha \left( {{t_{j + 1}} - {{{\tilde{t}}}_j}} \right) {{\bar{V}}_i}({t_{j + 1}}) < \alpha {\varpi _j}\left( {{t_{j + 1}} - {t_j}} \right) {e^{\alpha {\varpi _j}({t_{j + 1}} - {t_j})}}{{\bar{V}}_i}({{\tilde{t}}_j}). \end{aligned}$$

Due to $\beta \left( {{{{\tilde{t}}}_j} - {t_j}} \right) {{\bar{V}}_i}({{\tilde{t}}_j}) = \beta \left( {1 - {\varpi _j}} \right) \left( {{t_{j + 1}} - {t_j}} \right) {{\bar{V}}_i}({{\tilde{t}}_j})$, it can be revealed via (43) that

44 $$\begin{aligned} \alpha \left( {{t_{j + 1}} - {{{\tilde{t}}}_j}} \right) {{\bar{V}}_i}({t_{j + 1}}) - \beta \left( {{{{\tilde{t}}}_j} - {t_j}} \right) {{\bar{V}}_i}({{\tilde{t}}_j}) < \left( {\alpha {\varpi _j}{e^{\alpha {\varpi _j}({t_{j + 1}} - {t_j})}} - \beta \left( {1 - {\varpi _j}} \right) } \right) {{\bar{V}}_i}({{\tilde{t}}_j})\left( {{t_{j + 1}} - {t_j}} \right) . \end{aligned}$$

If $\beta (1 - {\tilde{\varpi }} ) > \alpha {\tilde{\varpi }} {e^{\alpha {\tilde{\varpi }} {t_{\max }}}}$, then one can obtain from (44) that

$$\begin{aligned} \sum \limits _{i = 2}^M {\sum \limits _{j = 0}^{ + \infty } {\left( {\alpha \left( {{t_{j + 1}} - {{{\tilde{t}}}_j}} \right) {{{\bar{V}}}_i}({t_{j + 1}}) - \beta \left( {{{{\tilde{t}}}_j} - {t_j}} \right) {{{\bar{V}}}_i}({{{\tilde{t}}}_j})} \right) } } < 0. \end{aligned}$$

Hence, one can obtain by (42) that $E \leqslant \sum \nolimits _{i = 2}^M {{{{\bar{V}}}_i}(0)} .$ The rest proof is similar to Theorem 1. $\square$

Moreover, for any given gain matrix *K*, the following corollary can be obtained, which shows a sufficient condition of minimum-energy-restriction formation analysis for interconnected system (1) with formation protocol (P2), where the interaction silence is considered.

### Corollary 3

For any given *K*, interconnected system (1) with formation protocol (P2) achieves minimum-energy-restriction formation if $A{\xi _i}(t) = {\dot{\xi }_i}(t)$ $\left( {i = 1,2, \ldots ,M} \right)$ and there exists $\alpha > 0,$ $\beta > 0$ and ${{\hat{P}}^T} = {\hat{P}} > 0$ such that $\beta (1 - {\tilde{\varpi }} ) > \alpha {\tilde{\varpi }} {e^{\alpha {\tilde{\varpi }} {t_{\max }}}}$ and the following minimization problem has an optimal solution $\eta$:

$$\begin{aligned} \hbox {min} & \quad \eta \\ \hbox {s. t.} & \quad {{\hat{\Omega }} _1} = {\hat{P}}A + {A^T}{\hat{P}} - \alpha {\hat{P}}< 0,\\ & \quad {\hat{\Omega }} _2^i = \left[ {\begin{array}{*{20}{c}} {{\hat{P}}A + {A^T}{\hat{P}} - {\lambda _i}{K^T}{B^T}{\hat{P}} - {\lambda _i}{\hat{P}}BK + \beta {\hat{P}}}&{{\lambda _i}{K^T}W} \\ * &{ - W} \end{array}} \right]< 0\,\,\left( {i = 2,M} \right) ,\\ & \quad {{\hat{\Omega }} _3} = {\hat{P}} - \eta {I_d} < 0. \end{aligned}$$

### Remark 3

The effects of the interaction silence among agents on the minimum-energy-restriction formation mainly contain two aspects. The first aspect is that the cooperative state may diverge away during the interaction silence subsegment for the unstable system matrix since there does not exist any control action. The inequality condition $\beta (1 - {\tilde{\varpi }} ) > \alpha {\tilde{\varpi }} {e^{\alpha {\tilde{\varpi }} {t_{\max }}}}$ is imposed, which guarantees that the divergent quantity during the interaction silence subsegment is less than the convergent quantity during the information non-silence subsegment by choosing two inhibition parameters $\alpha$ and $\beta$ properly. In addition, the convergent quantity is affected by the silence rate ${\varpi _j}\mathrm{{ }}\left( {j \in {\bar{\mathbb {Z}}^ - }} \right)$. When the dynamics of agents are not Lyapunov stable, the relative dynamics of the interconnected system will diverge away if the silence rate is too large. The second aspect is to constrain the effects of the interaction silence on the energy restriction. By introducing the term ${e^{\alpha {\tilde{\varpi }} {t_{\max }}}}$ in the inequality condition $\beta (1 - {\tilde{\varpi }} ) > \alpha {\tilde{\varpi }} {e^{\alpha {\tilde{\varpi }} {t_{\max }}}}$, the energy restriction is bounded by the sum of the Lyapunov functional candidates at time zero. Especially, if the energy restriction is neglected, then this inequality condition can be simplified as $\beta (1 - {\tilde{\varpi }} ) > \alpha {\tilde{\varpi }}$, which can handle the impact of the interaction silence. Intuitionally speaking, the optimal solution of Theorems 1 and 2 can be obtained earlier if the upper bound of the interaction silence rate is smaller. Moreover, it can be found by (25) that the interaction silence does not impact the formation whole motion trajectory when minimum-energy-restriction formation is achieved, but it is critically important for interconnected system (1) with formation protocol (P2) to achieve minimum-energy-restriction formation.

## Simulation examples

The following examples are proposed to reveal the validness of conclusions about the minimum-energy-restriction formation with and without the interaction silence, respectively. Consider a water-floating platform as the physical motion system model, the control target of the agents in the water-floating platform is to keep the platform horizontal. Since the waves are disturbing the platform all the time, the whole motion trajectory of all agents is time-varying. Moreover, it may be necessary to maintain a certain state value between different platforms, so the formation control case of water-floating platforms is taken into account.

### Example 1

**Figure 1:**
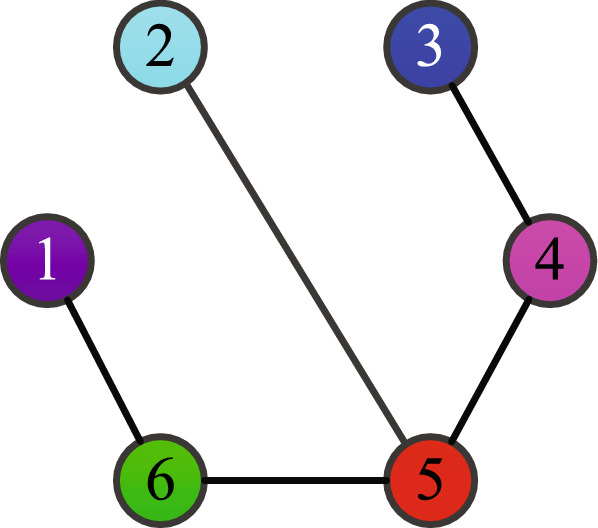
Information topology.

(*Minimum-energy-restriction formation without interaction silence*): Consider six agents communicating with each other by the information topology showing in Fig. 1. If agent *i* can receive the state information of agent *k* directly, then the channel strength ${w_{ik}} = 1$ $\left( {\forall i,k \in \left\{ {1,2, \ldots ,6} \right\} {\text { and }}i \ne k} \right)$, and ${w_{ik}} = 0$ otherwise.

The dynamic matrices are designed as:

$$\begin{aligned} A = \left[ {\begin{array}{*{20}{c}} 0&0&{ - 1} \\ { - 3}&{ - 3}&1 \\ { - 421}&{ - 422}&0 \end{array}} \right] ,\,\,B = \left[ {\begin{array}{*{20}{l}} {0.6}&3 \\ 6&0 \\ 0&3 \end{array}} \right] . \end{aligned}$$

The initial states are set as:

$$\begin{aligned} {x_1}(0)&= {\left[ { - 30,5, - 15} \right] ^T},{x_2}(0) = {\left[ {5,6, - 20} \right] ^T}, \\ {x_3}(0)&= {\left[ { - 18,1,30} \right] ^T},\,\,{x_4}(0) = {\left[ {12,2, - 1} \right] ^T}, \\ {x_5}(0)&= {\left[ { - 5,1.5,10} \right] ^T},\,\,{x_6}(0) = {\left[ { - 10,1, - 15} \right] ^T}. \\ \end{aligned}$$

The time-varying formation control vectors are described by:

$$\begin{aligned} {\xi _i}(t) = \left[ {\begin{array}{*{20}{c}} {70\sin \left( {8t + \frac{{\left( {i - 1} \right) \pi }}{3}} \right) } \\ { - 70\sin \left( {8t + \frac{{\left( {i - 1} \right) \pi }}{3}} \right) } \\ { - 70\cos \left( {8t + \frac{{\left( {i - 1} \right) \pi }}{3}} \right) } \end{array}} \right] \,\,\left( {i = 1,2, \ldots ,6} \right) , \end{aligned}$$

which satisfy $A{\xi _i}(t) = {\dot{\xi }_i}(t)\,\,\left( {i = 1,2, \ldots ,6} \right)$. When $\xi (t)$ is realized, the states of six agents form a time-varying equilateral hexagon formation and rotate around the center of six agents.

Let $W = {\text {diag}}\left\{ {0.006,0.006} \right\}$, then the matrix variable in Theorem 1 can be obtained by the GEVP solver as:

$$\begin{aligned} P = \left[ {\begin{array}{*{20}{c}} {0.1985}&{ - 0.1997}&{0.0819} \\ { - 0.1997}&{0.2014}&{ - 0.0590} \\ {0.0819}&{ - 0.0590}&{2.2836} \end{array}} \right] \times {10^5}. \end{aligned}$$

In this case, $\eta = 0.1306.$ Then, *K* can be obtained as:

$$\begin{aligned} K = \lambda _2^{ - 1}{B^T}{P^{ - 1}} = \left[ {\begin{array}{*{20}{l}} {1.0846}&{1.0733}&{ - 0.0112} \\ {0.4929}&{0.4874}&{ - 0.0051} \end{array}} \right] . \end{aligned}$$

In Theorem 1, when ${\Omega _2} = {I_d} - \eta P < 0$ is removed, the conclusions are converted into the formation criterion for the interconnected system without the minimum energy restriction. To distinguish the calculation process with and without the minimum energy restriction, *P* and *K* are denoted by ${P_1}$ and ${K_1}$. Let $E_1^{\min }(t)$ and ${E_1}(t)$ denote the total energy restriction of the interconnected system with and without the minimum energy restriction, respectively. ${P_1}$ and ${K_1}$ can be solved by the FEASP solver as:

$$\begin{aligned} {P_1}&= \left[ {\begin{array}{*{20}{c}} {206.5732}&{ - 206.9622}&{72.0020} \\ { - 206.9622}&{207.6703}&{ - 71.1372} \\ {72.0020}&{ - 71.1372}&{395.4840} \end{array}} \right] , \\ {K_1}&= \lambda _2^{ - 1}{B^T}P_1^{ - 1} = \left[ {\begin{array}{*{20}{l}} {54.9367}&{54.7738}&{ - 0.1494} \\ {24.9974}&{24.8939}&{ - 0.0534} \end{array}} \right] . \end{aligned}$$

**Figure 2:**
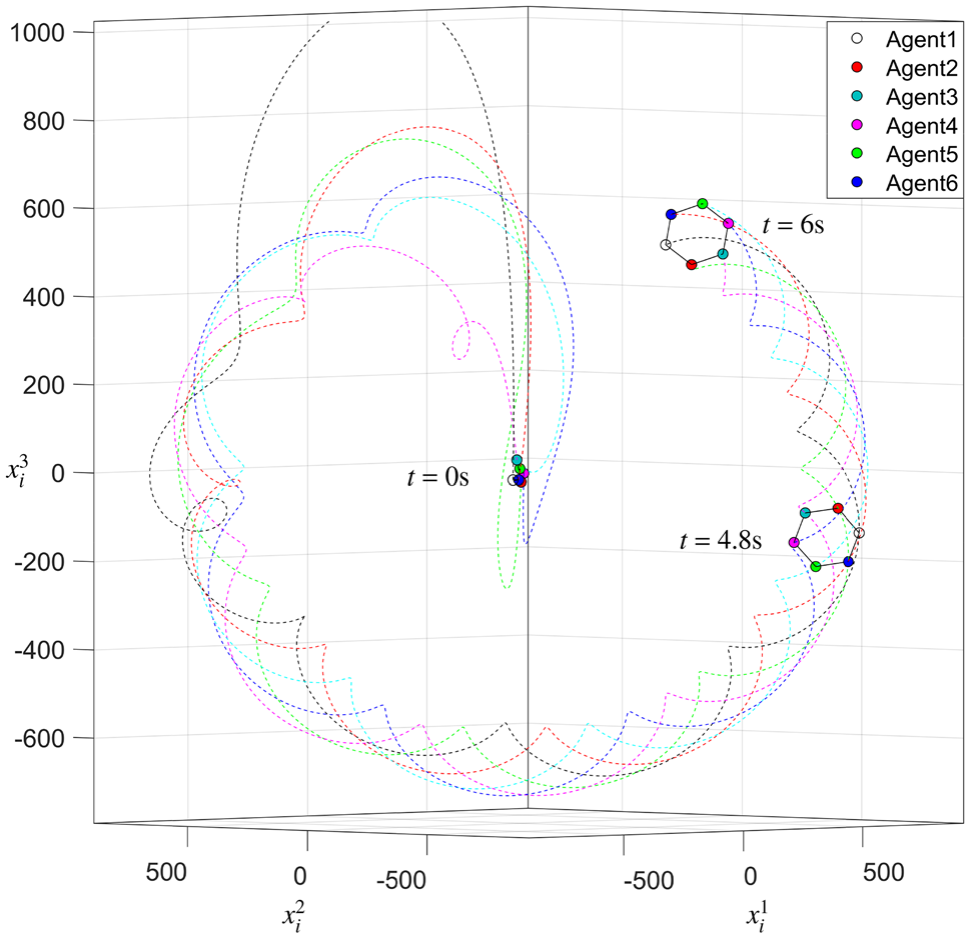
State trajectories of all agents without interaction silence.

**Figure 3:**
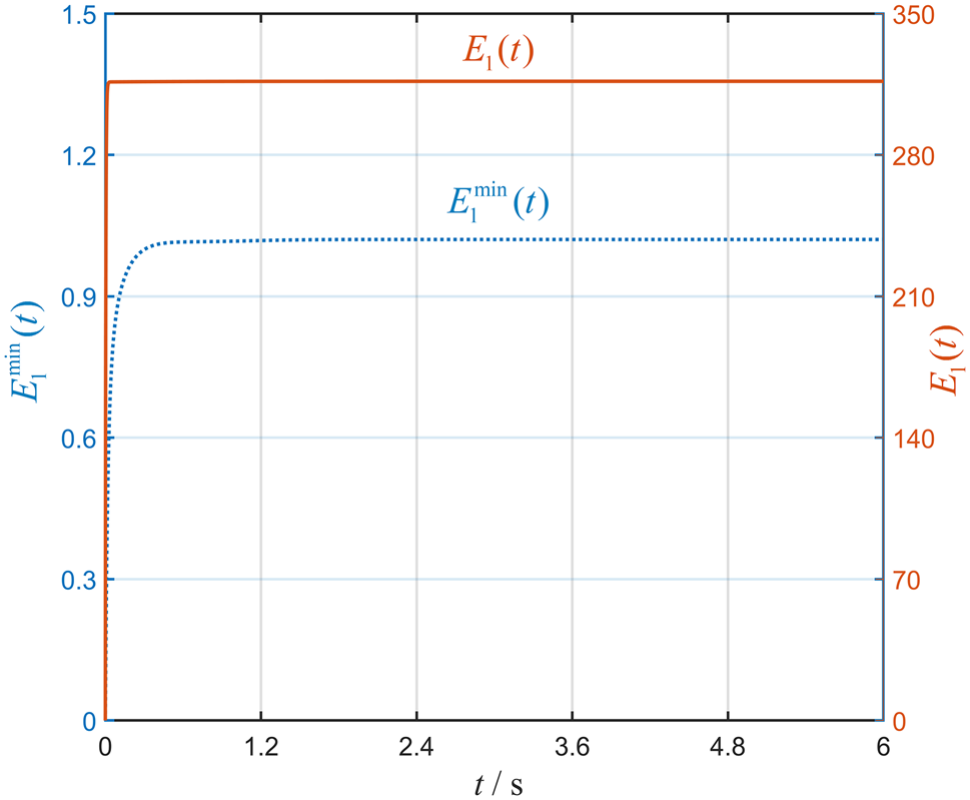
Curves of $E_1^{\min }(t)$ and ${E_1}(t)$.

With the minimum energy restriction, the state trajectories of interconnected system (1) within $6{\text {s}}$ are illustrated in Fig. 2. The states of various agents are shown with six different colored circles, and the desired equilateral hexagon formation is achieved. Simultaneously, the states of six agents are rotating around the center of all agents, which means that the hexagon formation is time-varying. The total energy restrictions $E_1^{\min }(t)$ and ${E_1}(t)$ in Fig. 3 converge to a finite value. One can conclude that interconnected system (1) is minimum-energy-restriction formation achievable by formation protocol (P1) from Figs. 2 and 3. Furthermore, we can find that the total energy restriction with the minimum energy restriction is less than the one without the minimum energy restriction; that is, $E_1^{\min }(t) < {E_1}(t)$.

### Example 2

(*Minimum-energy-restriction formation with interaction silence*): The information topology of this interconnected system with the interaction silence is also depicted in Fig. 1. The system matrices for each agent are designed as:

$$\begin{aligned} A = \left[ {\begin{array}{*{20}{c}} 0&0&{ - 1} \\ { - 1}&{ - 1}&1 \\ { - 2}&{ - 3}&0 \end{array}} \right] ,\,\,B = \left[ {\begin{array}{*{20}{c}} { - 1}&1 \\ 1&0 \\ {0.5}&1 \end{array}} \right] . \end{aligned}$$

The initial states and formation control vectors are set as:

$$\begin{aligned} & {x_1}(0) = {\left[ {1,1, - 1} \right] ^T},{{ }}{x_2}(0) = {\left[ {9,6, - 2} \right] ^T}, \\ & {x_3}(0)= {\left[ { - 1,1,0} \right] ^T},{{ }}{x_4}(0) = {\left[ {1,2, - 1} \right] ^T}, \\ & {x_5}(0) = {\left[ { - 2,0,1} \right] ^T},{{ }}{x_6}(0) = {\left[ { - 1,1, - 1} \right] ^T}, \\ & {\xi _i}(t) = \left[ {\begin{array}{*{20}{c}} {0.7\sin \left( {8t + \frac{{\left( {i - 1} \right) \pi }}{3}} \right) } \\ { - 0.7\sin \left( {8t + \frac{{\left( {i - 1} \right) \pi }}{3}} \right) } \\ { - 0.7\cos \left( {8t + \frac{{\left( {i - 1} \right) \pi }}{3}} \right) } \end{array}} \right] \,\,\left( {i = 1,2, \ldots ,6} \right) . \end{aligned}$$

The silence subsegment and the non-silence subsegment are set as $t \in \left[ {{t_j} + 0.8,{t_j} + 1} \right) {\text {s }}\left( {\forall j \in {{\bar{\mathbb {Z}}}^ - }} \right)$ and $t \in \left[ {{t_j},{t_j} + 0.8} \right) {\text {s}}$ with ${t_0} = 0{\text {s}}$, respectively, then it can be found that the upper bound of the silence rate is ${\tilde{\varpi }} = 0.2$. Let $\alpha = 2,$ $\beta = 0.9$ and $W = {\text {diag}}\left\{ {0.006,0.006} \right\}$, then it can be found that $\beta (1 - {\tilde{\varpi }} ) > \alpha {\tilde{\varpi }} {e^{\alpha {\tilde{\varpi }} {t_{\max }}}}$ is satisfied. By utilizing the GEVP solver, the matrix variable ${\bar{P}}$ in Theorem 2 can be calculated as:

$$\begin{aligned} {\bar{P}} = \left[ {\begin{array}{*{20}{c}} {4.8428}&{ - 1.6142}&{4.8427} \\ { - 1.6142}&{0.5381}&{ - 1.6142} \\ {4.8427}&{ - 1.6142}&{4.8426} \end{array}} \right] \times {10^6}. \end{aligned}$$

In this case, $\eta = 7.7396.$ Then

$$\begin{aligned} K = \lambda _2^{ - 1}{B^T}{{\bar{P}}^{ - 1}} = \left[ {\begin{array}{*{20}{c}} {0.1401}&{2.3219}&{0.6338} \\ {2.7944}&{4.2562}&{ - 1.3757} \end{array}} \right] . \end{aligned}$$

We consider the distributed formation control with and without the minimum energy restriction where ${{\bar{\Omega }} _3} = {I_d} - \eta {\bar{P}} < 0$ is removed when the minimum energy restriction is not taken into account. Denoted ${\bar{P}}$ and *K* by ${{\bar{P}}_2}$ and ${K_2}$ to distinguish above cases. When there exists the interaction silence, the total energy restrictions of the interconnected network with and without the minimum-energy-restriction are denoted by $E_2^{\min }(t)$ and ${E_2}(t)$, respectively. Then, ${{\bar{P}}_2}$ and ${K_2}$ can be obtained as:

$$\begin{aligned} {{\bar{P}}_2}&= \left[ {\begin{array}{*{20}{c}} {21.2534}&{ - 7.1451}&{21.3158} \\ { - 7.1451}&{2.8228}&{ - 6.3571} \\ {21.3158}&{ - 6.3571}&{23.8885} \end{array}} \right] , \\ {K_2}&= \lambda _2^{ - 1}{B^T}{\bar{P}}_2^{ - 1} = \left[ {\begin{array}{*{20}{c}} {0.2409}&{2.9362}&{0.6212} \\ {3.7638}&{5.5164}&{ - 1.7809} \end{array}} \right] . \end{aligned}$$

**Figure 4:**
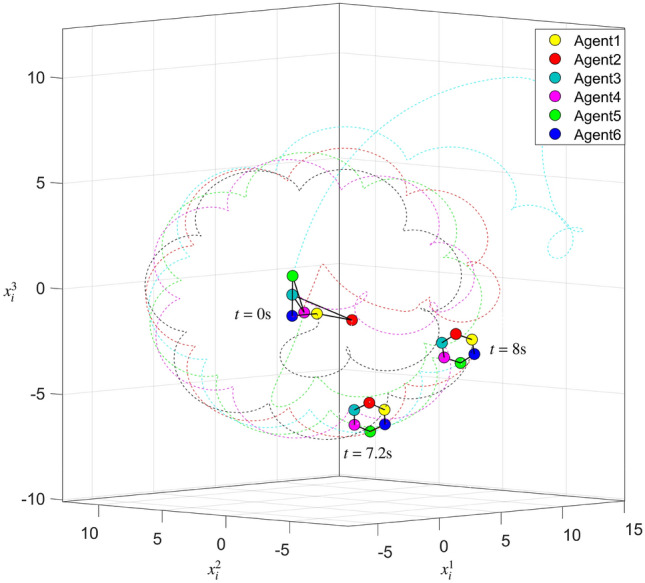
State trajectories of all agents with interaction silence.

**Figure 5:**
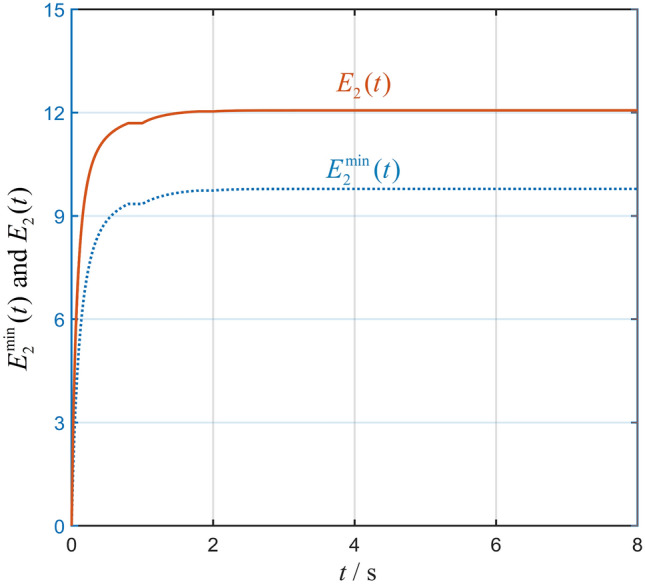
Curves of $E_2^{\min }(t)$ and ${E_2}(t)$.

With the non-periodic interaction silence and the minimum-energy-restriction, the states of six agents within $8{\text {s}}$ are shown in Fig. 4 by six color dotted curves. We can find that the desired hexagram formation is achieved. In Fig. 5, the curves of $E_2^{\min }(t)$ and ${E_2}(t)$ with $t \in \left[ {0,8} \right] {\text {s}}$ are shown, and one can reach a conclusion that interconnected system (1) is minimum-energy-restriction formation achievable by formation protocol (P2) with the interaction silence. Furthermore, it can be found that the total energy restriction without the minimum energy restriction is more than the total energy restriction with the minimum energy restriction.

## Conclusions

With the total energy restriction and the interaction silence, a distributed formation protocol for interconnected systems was proposed to realize formation control under the condition that the total energy restriction is minimum. By constructing a common Lyapunov functional candidate, sufficient conditions for minimum-energy-restriction formation of interconnected systems without the interaction silence were proposed. This method ensures that the energy restriction is minimum and can be solved efficiently using the generalized eigenvalue methods. Moreover, an explicit expression of the formation whole motion trajectory was given, which contains two independent parts: the trajectory associated with the average of the initial states of all agents and the trajectory associated with the average of formation control vectors of all agents. Furthermore, the impacts of the interaction silence on minimum-energy-restriction formation were determined by introducing two inhibition parameters and the interaction silence rate, where it is essentially required that the convergent quantity during the information non-silence subsegment is larger than the divergent quantity during the interaction silence subsegment.

## Data Availability

The datasets used and/or analysed during the current study available from the corresponding author on reasonable request.
